# The Combined Measurement of Pelvic Organ Mobility and Hiatus Area Improves the Sensitivity of Transperineal Ultrasound When Detecting Pelvic Organ Prolapse

**DOI:** 10.3389/fmed.2021.727711

**Published:** 2021-10-27

**Authors:** Xiaoduo Wen, Haiyan Tian, Xiaojing Yan, Quiqing Sun, Yuanyuan Du, Denggui Wen, Yi Yang

**Affiliations:** ^1^Department of Ultrasound in Obstetrics and Gynecology, Fourth Hospital of Hebei Medical University, Shijiazhuang, China; ^2^Department of Medical Statistics, Fourth Hospital of Hebei Medical University, Shijiazhuang, China

**Keywords:** transperineal ultrasound, levator hiatus area, pelvic organ mobility, sensitivity of joint measurement, pelvic organ prolapse

## Abstract

**Objective:** To evaluate whether the combined measurement of pelvic organ mobility and levator hiatus area improves the sensitivity of transperineal ultrasound (the index test) for diagnosing pelvic organ prolapse (POP).

**Methods:** We retrospectively recruited women who had been examined in a tertiary gynecological center for symptoms of lower urinary tract incontinence and/or POP between January 2017 and June 2018. We excluded patients who had undergone hysterectomy previously or those who had received corrective surgery. All subjects underwent a standardized interview, POP quantification (POP-Q) examination (a reference standard for patients and controls), and ultrasound measurements of the levator hiatus area at rest (rHA), on contraction (cHA), and on Valsalva (vHA). We also determined the mobility of the bladder neck (BNM), cervix (CM), and rectum ampulla (RAM). Receiver operating characteristic (ROC) curve analyses were performed to determine cut-off values for diagnosis. Diagnostic performance was assessed by sensitivity, specificity, and area under curve (AUC).

**Results:** A total of 343 women were eligible for analysis, including 247 POP patients (stage 2–3 by POP-Q) and 96 controls. Compared with controls, POP cases had significantly higher values for rHA, vHA, cHA, BNM, CM, and RAM. Each parameter was identified as a significant discriminator for POP and controls, as determined by ROC curve analysis, although the cut-off value varied slightly between different parameters. The combination of rHA, vHA, and cHA (with any HA that was ≥ the cut-off) improved the sensitivity from 64–89 to 89–93%. The combination of pelvic organ mobility with rHA, vHA, and cHA, further increased the sensitivity from 89–93 to 95–97%.

**Conclusion:** The combination of levator hiatus area and pelvic organ mobility improved the sensitivity of transperineal ultrasound in the diagnosis of POP, whether used as a frontline test to assist POP-Q grading or to monitor the effect of pelvic floor exercise programs.

## Introduction

The prevalence of pelvic organ prolapse (POP) in women has increased steadily as human longevity has increased. This condition compromises the life quality of millions of adult women worldwide and has become a major health burden (1, 2). The quantification of POP is pivotal for optimal patient management (3). The International Continence Society Pelvic Organ Prolapse Quantification (ICS POP-Q) system was first introduced in 1996 and is now regarded as the most reliable grading system (4–6). However, the POP-Q system only provides information relating to the surface anatomy of the pelvic organs and does not consider underlying organs or functional anatomy (3). POP is characterized by ballooning of the levator hiatus and the protrusion of pelvic organs *via* a weakened pelvic floor (3, 7, 8). Transperineal ultrasound can detect both of these inner alterations and yields reproducible results (9, 10). Most previous studies that addressed the diagnosis of POP by transperineal ultrasound have measured only one parameter, either levator hiatus area or pelvic organ mobility (8, 11–14). We hypothesized that the combined measurement of levator hiatus area and pelvic organ mobility may increase the sensitivity of transperineal ultrasound for the diagnosis of POP.

## Materials and Methods

This prospective study recruited women who were examined in the Urogynecological Clinic in the Department of Obstetrics & gynecology at a tertiary Medical Center between January 2017 and June 2018 for pelvic floor disorder symptoms such as vaginal bulge and/or stress urinary incontinence (SUI). The study was approved by the Institutional Ethics Review Board of the Fourth Hospital of Hebei Medical University (Reference: 20160203). A consent form was signed by all participants.

Women included in the study all underwent a standardized clinical interview, clinical examination using the POP-Q system, and two-dimensional (2D) and three-dimensional (3D) transperineal ultrasound examination to determine the mobility of the bladder neck (BNM), cervix (CM), rectum ampulla (RAM); we also used these techniques to determine the levator hiatus area at rest (rHA), on maximal contraction (cHA), and on Valsalva maneuver (vHA). The exclusion criteria were as follows: (a) women with a history of hysterectomy or pelvic floor surgery for prolapse/incontinence; (b) women who failed to perform an adequate Valsalva maneuver (5 seconds or longer), and (c) women <6 months postpartum [this was because postpartum women represent a very different population]. We also excluded women with stage 4 or complete vaginal prolapse (irreducible at rest or contraction) because prolapse at this stage may interfere with transperineal ultrasound measurements.

### The POP-Q System

POP-Q examinations were performed by an experienced gynecologist (TH). As recommended, clinically significant prolapse was defined as POP-Q stage 2 or higher in the anterior and posterior compartments, and stage 1 or higher in the apical compartment (15).

### Transperineal Ultrasound Imaging

Two-dimensional (2D) and 3D transperineum ultrasound examinations were performed by two experienced ultrasonographers with ≥2 years of experience of the ultrasound POP examination (WX & YX) who were blinded to demographic and POP-Q data. All ultrasound examinations involved a Mindray Resona 8 Elite system (Shenzhen, P. R. China.) with a RAB 4–8 MHz curved array volume transducer. Women were placed in the supine position with flexed and slightly abducted hips with a urine volume <50 ml, as determined by ultrasound. The transducer was covered with a condom and placed on the perineum, in the mid-sagittal plane with the application of minimal pressure. The quality of pelvic floor muscle contractions was subjectively evaluated on a 2D cine loop obtained at rest and on contraction. Image acquisition was performed with the main axis of the transducer in the mid-sagittal plane, showing the inferior margin of the pubis, urethra, and bladder neck, as well as the levator ani muscle posterior to the anorectal junction. The preferred image orientation was with the symphysis pubis to the left and the anorectal canal to the right (3).

**Figure 1:**
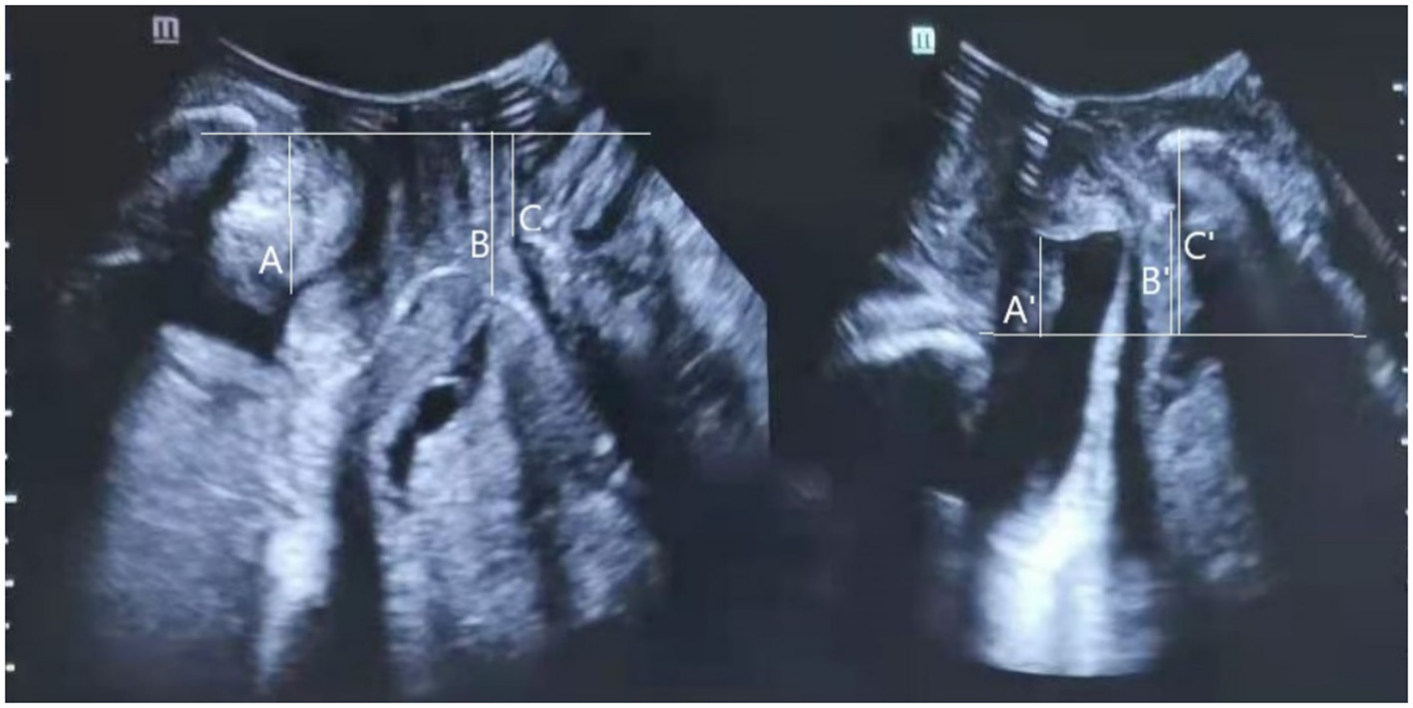
Transperineal ultrasound image in the mid-sagittal plane demonstrating the measurement of the distance between the leading edge of the bladder neck (A at rest; A' under Valsalva), cervix (B at rest; B' under Valsalva), or rectal ampulla (C at rest; C' under Valsalva), and the horizon line at the level of the posterior inferior margin of the pubic symphysis. Measurements cranial to the reference line are positive; those caudal to the line are negative. Bladder neck mobility (BNM), cervix mobility (CM), and rectum ampulla mobility (RAM), were calculated as the absolute difference between rest and Valsalva maneuver in the pubis-bladder neck, pubis-cervix, or pubis-rectum ampulla distance.

Pelvic organ descent was measured in relation to the posterior-inferior margin of the pubis in the midsagittal plane, referencing the vertical distance between the symphysis pubis and bladder neck, cervix, and the rectum ampulla (16). Measurements inside the posterior-inferior pubic margin were defined as negative values while measurements outside were defined as positive values (Figure 1) (17). The absolute difference between rest and the Valsalva maneuver in the vertical pubis-pelvic organ distance was calculated to represent the mobility of the pelvic organ.

**Figure 2:**
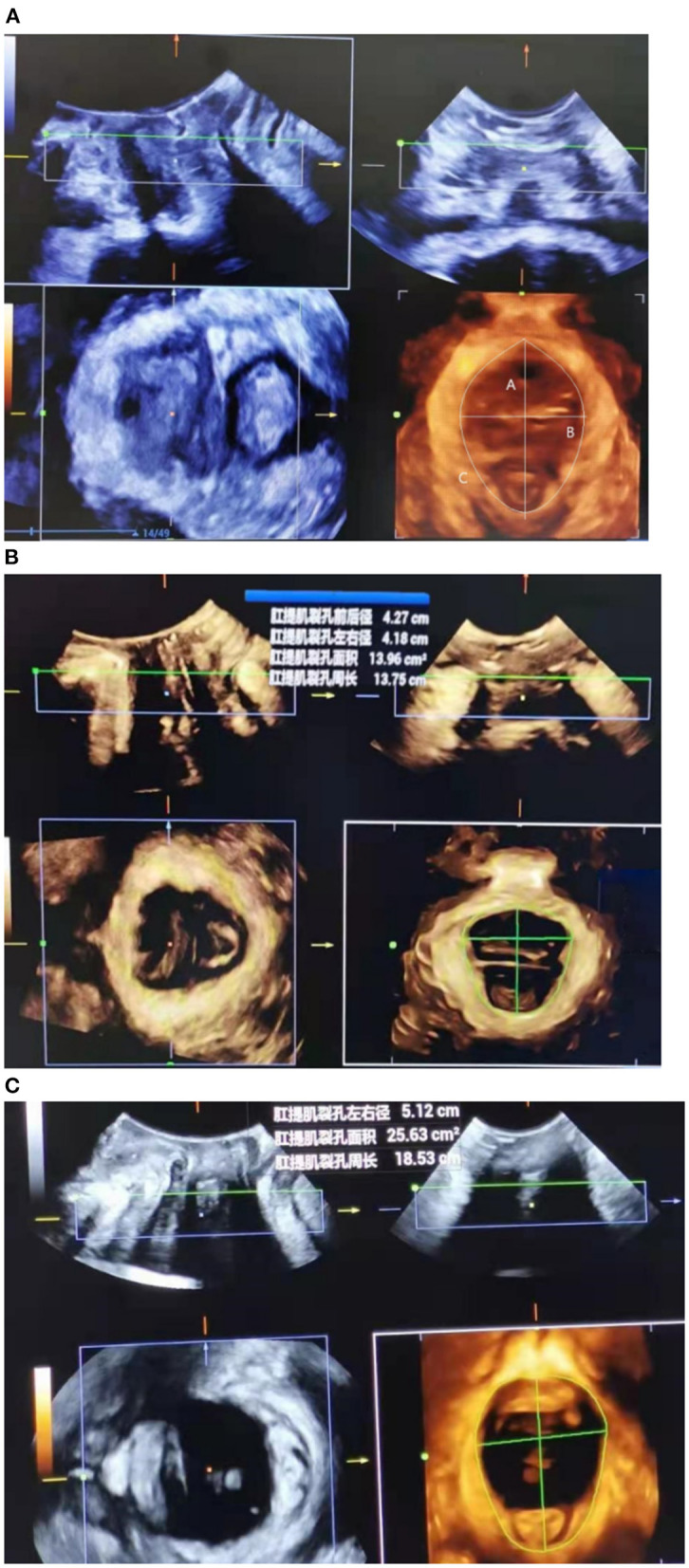
Transperineal ultrasound image demonstrating the measurement of levator hiatal anteroposterior diameter (upper left), left-right diameter (upper right), and area (lower left) at rest **(A)**, at maximal contraction **(B)**, or under Valsalva maneuver **(C)**.

Then, 3D ultrasound volumes were acquired with the acquisition angle set to 85° or higher, thus allowing visualization of the entire levator hiatus area. The hiatal area was measured at rest, on Valsalva maneuver, and at maximum contraction (Figure 2).

The technique used for acquiring three dimensional imaging was the same as described for obtaining two dimensional imaging in maximum contraction. The inferior margin of the symphysis pubis was used as a reference point in two- and three dimensional datasets for identification of Levator ani avulsion. Tomographic ultrasound imaging (TUI) was used for quantification of levator defects. A set of 8 slices with an interslice interval of 2.5 mm was obtained, from 5.0 mm below to 12.5 mm above the hiatal plane, in a volume obtained on maximal levator contraction. Levator “avulsion” was defined as a clear detachment of the muscle anteromedial from the pubic bone during contraction in any of the three central slices, either unilateral or bilateral.

### Statistical Analysis

The SPSS statistical package, version 25.0 for Windows (IBM, Armonk, NY, USA) was used to perform statistical analyses. rHA, vHA, cHA, BNM, CM, and RAM, are expressed as mean ± standard deviation (SD). The Pearson correlation coefficient between any two of the above six ultrasound measurement was calculated. The distribution of continuous data was assessed using the Kolmogorov–Smirnov test. Mean values were compared between compartment predominant prolapse and controls (as defined by POP-Q) using the student's t-test when normally distributed or the Mann–Whitney U-test when the distribution was not normal. Statistical significance was considered at *P* < 0.05. When ultrasound parameters were found to be significantly different when compared between prolapse cases and controls, we constructed receiver operating characteristics (ROC) curves to determine the optimal cut-off values and to show the performance of the levator hiatus area at rest (rHA), under Valsalva maneuver (vHA), and at maximal contraction (cHA), and the performance of bladder neck mobility (BNM), cervix mobility (CM), and rectum ampulla mobility (RAM) from rest to Valsalva, separately or in combination, for discriminating between women with and without compartment predominant prolapse (International Continence Society POP quantification, stage 2–3). When constructing ROC curves, age (under or ≥50 years older) and body mass index (BMI) (under 25 kg/m^2^ or larger) were included as adjust variables Finally, the three HAs and the compartment-specific mobility measurements were combined to improve the sensitivity of the discriminatory test. Sensitivity and specificity were defined as the predictive ability of true positivity of the prolapse patients, and the true negativity of the controls, respectively. Positive predictive value (PPV), negative predictive value (NPV), and 1-NPV were defined as the percentages of patients among the positive ultrasound results, the percentage of controls among the negative ultrasound results, or the percentage of women with normal hiatal area value concomitant with grade 2–3 cystocele, uterine or rectal prolapse).

## Results

Of the 493 women recruited, 80 had undergone pelvic floor surgery previously. Of these, 30 had a history of hysterectomy, 21 had stage 4 prolapse, and 19 failed to perform an adequate Valsalva maneuver; consequently, these women were excluded prior to ultrasound. The remaining 343 women constituted the final study sample; there were 247 (72%) stage 2–3 POP patients and 96 (28%) controls (Table 1).

**Table 1:** Demographic and reproductive history characteristics of 343 women visiting a urogynecology clinic according to the diagnosis of significantly objective prolapse (International Continence Society POP quantification, stage 2–3).

**Characteristic**	**POP-Q stage 2–3** ***n (%)***	**Control** ***n (%)***	***P*-value**
Age (yrs.) (X ± S)	46.44 ± 5.62	36.39 ± 11.17	<0.001
BMI (X ± S)	26.14 ± 2.96	24.00 ± 3.07	<0.001
Parity (n, %)			
Nullipara	0 (0)	4 (4.2)	<0.001
Primipara	92 (37.2)	63 (65.6)	
Multipara	155 (62.8)	29 (30.2)	
Delivery method			
Nulliparous	0 (0)	4 (4.2)	<0.001
Cesarean section	21 (8.5)	39(40.6)	
Virginal delivery	226 (91.5)	53(55.2)	
Levator avulsion by TUI^*^	73(29.6)	5(5.2)	<0.001
Compartment of POP			
None		96 (100)	
Anterior	89 (36.03)		
Apical	19 (7.69%)		
Posterior	7 (2.83)		
Anteroapical	94 (38.06)		
Anteroposterior	12 (4.86)		
Apicoposterior	6 (2.43)		
Anteroapicoposterior	20 (8.10)		

TUI* *Tomographic ultrasound imaging*.

The prolapse cases were significantly older and had a significantly larger body mass index (BMI) than the controls (mean age 46.44 vs. 36.39 years, *P* < 0.001; BMI 26.14 vs. 24.00 kg/m^2^, *P* < 0.001). Compared with controls, significantly more women with prolapse had experienced multiple births (62.8% *vs*. 30.2%, *P* < 0.001), gave birth by vaginal delivery (91.5 vs. 55.2%, *P* < 0.001), and had levator avulsion as detected by Tomographic Ultrasound Imaging (TUL) (29.6 vs. 5.2%, *P* < 0.001).

With regards to the prolapse compartment, the proportions of singular compartments involved were 36.03, 7.69, and 2.83%, for the anterior, apical, and posterior, respectively. The proportions of multiple compartment involvement (anteroapical, anteroposterior, apicoposterior, or anteroapicoposterior) were 38.06%, 4.86%, 2.43, and 8.1%, respectively (Table 1). For analysis, the compartment with the most severe prolapse determined the designation of the predominant compartment.

A test-retest series, involving a total of 25 patients, was performed by two operators (WXD and YXJ) who both analyzed the same ultrasound images. The intra-class correlation coefficients (ICCs) for the inter-rater agreement for detecting the most caudad edges of the bladder neck, cervix and rectal ampulla, were 0.88 (95% confidence interval [CI]: 0.80–0.95), 0.81 (95% CI: 0.71–0.90) and 0.78 (95% CI: 0.74–0.95), respectively. The ICC was 0.87 for rHA (95% CI: 0.80–0.94), 0.90 for vHA (95% CI: 0.87–0.96), and 0.90 for cHA (95% CI: 0.84–0.98).

Significant correlation between ultrasound parameters was observed more often among POP-Q stage 2–3 patients than among the control group. Of the six ultrasound parameters, the Pearson correlation coefficient was significant only between BNM and any other parameter in the control group. However, the coefficient was significant between all parameters in the anterior predominant POP, between rHA, vHA, or CM and any other parameter in the apical predominant POP, and between rHA, CM, or RAM and any other parameter in the posterior predominant group (Table 2).

**Table 2:** Pearson correlation coefficient between any two of six ultrasound measurement of hiatal area (HA) at reast (rHA), on Valsalva (vHA), and on maximum contraction (cHA) and bladder neck mobility (BNM), cervix mobility (CM), and rectum ampulla mobility (RAM).

**Ultrasound measurement**	**rHA**	**vHA**	**cHA**	**BNM**	**CM**	**RAM**
Control						
rHA	1	0.74^**^	0.79^**^	0.234^*^	0.00	0.16
vHA	0.74^**^	1	0.82^**^	0.45^**^	0.09	0.16
cHA	0.79^**^	0.82^**^	1	0.37^**^	0.03	0.37^**^
BNM	0.23^*^	0.45^**^	0.37^**^	1	0.32^**^	0.38^**^
CM	0.00	0.09	0.03	0.32^**^	1	0.04
RAM	0.16	0.16	0.37^**^	0.38^**^	0.04	1
POP-Q stage 2–3 anterior predominant						
rHA	1	0.68^**^	0.66^**^	0.37^**^	0.40^**^	0.28^**^
vHA	0.68^**^	1	0.65^**^	0.56^**^	0.50^**^	0.36^**^
cHA	0.66^**^	0.65^**^	1	0.46^**^	0.49^**^	0.31^**^
BNM	0.37^**^	0.56^**^	0.46^**^	1	0.51^**^	0.29^**^
CM	0.40^**^	0.50^**^	0.49^**^	0.51^**^	1	0.34^**^
RAM	0.28^**^	0.36^**^	0.31^**^	0.29^**^	0.34^**^	1
POP-Q stage 2–3 apical predominant						
rHA	1	0.69^**^	0.61^**^	0.27^**^	0.37^**^	0.17^*^
vHA	0.69^**^	1	0.65^**^	0.45^**^	0.48^**^	0.22^*^
cHA	0.61^**^	0.65^**^	1	0.36^**^	0.44^**^	0.14
BNM	0.27^**^	0.45^**^	0.36^**^	1	0.45^**^	0.10
CM	0.37^**^	0.48^**^	0.44^**^	0.45^**^	1	0.27^**^
RAM	0.17^*^	0.22^*^	0.14	0.10	0.27^**^	1
POP-Q stage 2–3 posterior predominant						
rHA	1	0.53^**^	0.81^**^	0.35^*^	0.50^**^	0.48^**^
vHA	0.53^**^	1	0.54^**^	0.29	0.37^*^	0.52^**^
cHA	0.81^**^	0.54^**^	1	0.21	0.46^**^	0.42^**^
BNM	0.35^*^	0.29	0.21	1	0.58^**^	0.35^*^
CM	0.50^**^	0.37^*^	0.46^**^	0.58^**^	1	0.38^*^
RAM	0.48^**^	0.52^**^	0.42^**^	0.35^*^	0.38^*^	1

** *P < 0.01.*

* *P < 0.05*.

The mean values for rHA, vHA, and cHA were all significantly larger with stage 2–3 prolapse patients than the controls (All *P* < 0.01), but showed no significant differences across compartment predominant POP groups (Table 3). The optimum cut-off value for discriminating between each compartment-specific prolapse and controls was determined using ROC analysis separately for rHA, cHA, and vHA, in association with the sensitivity, specificity, area under curve (AUC), positive predictive value, negative predictive value, and accuracy etc.

**Table 3:** Comparison of levator hiatus area between women with and without compartment predominant prolapse (International Continence Society Pelvic Organ Prolapse Quantification (POP-Q), stage 2–3) at rest, maximal contraction, and under Valsalva maneuver.

**POP-Q/Compartment**	**N**	**Hiatal area (HA) (cm**^**2**^, ***$\bar{X}$*** **± S)**		
		**At rest**	**On valsalva**	**On contraction**
POP-Q stage 2 or higher combined	247	15.72 ± 3.39^**^	23.73 ± 6.71^**^	11.69 ± 3.63^**^
Compartment predominant prolapse				
Anterior predominant	215	15.80 ± 3.43^**^	23.94 ± 6.85^**^	11.70 ± 3.74^**^
Apical predominant	139	16.16 ± 3.58^**^	25.26 ± 6.80^**^	12.46 ± 3.68^**^
Posterior predominant	45	16.04 ± 3.89^**^	24.17 ± 6.52^**^	12.14 ± 3.69^**^
Control	96	12.96 ± 1.85	17.29 ± 3.35	8.30 ± 2.87

** *: P < 0.01*.

Although BNM, CM, and RAM were significantly larger in all of the prolapse groups than the controls, the largest BNM, CM, and RAM, were observed for the anterior-, apical-, and posterior, predominant prolapse cases, respectively (Table 4). Therefore BNM, CM, and RAM were used as the optimal parameters for discriminating between anterior, apical, or posterior predominant prolapse cases and controls in ROC analysis.

**Table 4:** Comparison of pelvic organ mobility (difference between rest and Valsalva in the distance from the symphysis pubis to the bladder neck (BNM), cervix (CM) and rectum ampulla (RAM), as measured by transperineal ultrasound) between control, prolapse, and compartment predominant prolapse (International Continence Society Pelvic Organ Prolapse Quantification (POP-Q), stage 2–3) patients.

**POP-Q/Compartment**	**N**	**Pelvic organ mobility (cm**^**2**^ ***$\bar{X}$*** **± S)**		
		**Bladder neck (BNM)**	**Cervix (CM)**	**Rectum ampulla (RAM)**
All prolapse cases combined	247	29.74 ± 12.55^**^	26.51 ± 16.30^**^	17.34 ± 11.28^**^
Compartment predominant prolapse cases				
Anterior predominant	215	31.60 ± 10.90^**^	26.90 ± 14.90^**^	17.40 ± 6.30^**^
Apicall predominant	139	29.90 ± 12.10^**^	30.30 ± 15.90^**^	18.40 ± 6.00^**^
Posterior predominant	45	28.70 ± 12.80^**^	26.30 ± 14.30^**^	20.60 ± 9.30^**^
Control	96	13.78 ± 8.97	13.08 ± 9.90	9.93 ± 6.37

** *: P < 0.01*.

**Figure 3:**
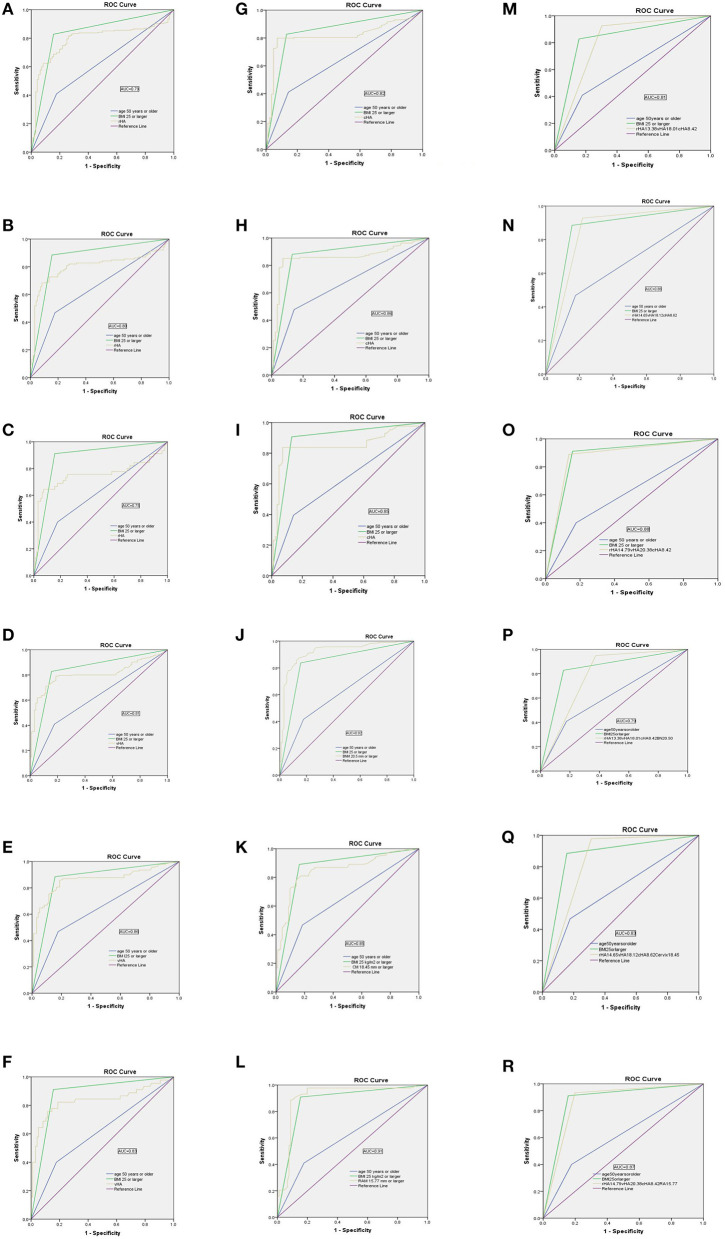
Receiver operator characteristic (ROC) curves showing the performance of the levator hiatus area at rest (rHA), under Valsalva (vHA), at maximal contraction (cHA), bladder neck mobility (BNM), cervix mobility (CM), and rectum ampulla mobility (RAM), separately or in combination, for discriminating between women with and without compartment predominant prolapse (POP-Q, stage 2–3). **(A–C)** rHA; **(D–F)** vHA; **(G–I)** cHA; **(J)** BNM; **(K)** CM; **(L)** RAM; **(M–O)** rHA+vHA+cHA; **(P)** rHA+vHA+cHA+BNM; **(Q)** rHA+vHA+cHA+CM; **(R)** rHA+vHA+cHA+RAM.

The optimum cut-off values for rHA with respect to predicting anterior-, apical-, or posterior predominant prolapse, were ≥13.38, ≥14.65, and ≥14.79 cm^2^; those of vHA for predicting corresponding prolapse groups were ≥18.01, ≥18.12, and ≥20.38 cm^2^; and those for cHA were ≥8.42, ≥8.62, and ≥8.42 cm^2^, respectively. rHA, vHA, and cHA had similar sensitivity, specificity, and AUC, irrespective of the compartment of prolapse being predicted (Table 5; Figures 3A–I).

**Table 5:** Receiver operating characteristic (ROC) analyses for the validity of using hiatal area at rest (rHA)/on Valsalva (vHA)/at contraction (cHA), and bladder neck mobility (BNM), cervix mobility (CM), and rectum ampulla mobility (RAM), either separately or in joint tests with transperineal ultrasound diagnosis of compartment predominant prolapse (International Continence Society POP quantification, grade II/ III).

**Parameter/Compartment predominant POP**	**N**	**Cut-off value**	**Sen (%)**	**Spe** **(%)**	**Area under curve (AUC)**	**Positive predictive value (PPV)** **(%)**	**Negative predictive value (NPV) (%)**	**1-NPV** **(%)**	***P***	**Accuracy (%)**
rHA (cm^2^)										
Anterior	215	≥13.38	81.4	74.0	0.79	87.5	64.0	36.0	0.00	79.11
Apical	139	≥14.65	68.3	91.7	0.80	92.2	66.7	33.3	0.00	77.86
Posterior	45	≥14.79	64.4	92.7	0.75	78.4	84.6	15.4	0.00	83.67
vHA (cm^2^)										
Anterior	215	≥18.01	79.1	81.3	0.81	90.4	63.4	36.6	0.00	79.80
Apical	139	≥18.12	85.6	81.3	0.86	86.9	79.6	20.4	0.00	83.90
Posterior	45	≥20.38	75.6	88.5	0.83	67.3	90.7	9.3	0.00	84.38
cHA (cm^2^)										
Anterior	215	≥8.42	79.5	83.3	0.82	91.0	64.2	35.8	0.00	80.07
Apical	139	≥8.62	84.9	83.3	0.86	87.4	79.0	21.0	0.00	84.24
Posterior	45	≥8.42	84.4	83.3	0.85	68.5	90.8	9.2	0.00	83.67
Pelvic organ mobility										
BNM anterior	215	≥20.50	85.6	87.5	0.92	93.8	72.4	27.6	0.00	86.20
CM apical	139	≥18.45	78.1	85.9	0.85	88.5	72.6	27.4	0.00	81.23
RAM posterior	45	≥15.77	88.6	91.2	0.91	68.9	96.3	3.7	0.00	90.35
rHA/vHA/cHA										
Anterior	215	≥13.38/18.01/8.42	92.6	69.8	0.81	87.3	80.7	19.3	0.00	85.50
Apical	139	≥ 14.65/18.12/8.62	92.8	78.1	0.86	85.4	88.1	11.9	0.00	86.81
Posterior	45	≥ 14.79/20.38/8.42	88.9	86.5	0.88	75.5	94.3	5.7	0.00	87.23
Combining rHA/vHA/cHA and compartment-specific pelvic organ mobility										
Anterior	215	≥ 13.38/18.01/8.42/20.50	94.9	62.5	0.79	85.0	84.5	15.5	0.00	84.89
Apical	139	≥ 14.65/18.12/8.62/18.45	97.1	63.1	0.83	81.9	95.7	4.3	0.00	82.98
Posterior	45	≥ 14.79/20.38/8.42/15.77	95.6	62.8	0.87	68.9	96.3	3.7	0.00	73.05

The optimum cut-off values for BNM, CM, and RAM, to predict anterior-, apical-, and posterior-predominant prolapse were ≥20.50 mm, ≥18.45 mm, and ≥15.77 mm, respectively: sensitivity/specificity/AUC values were 85.6%/97.5%/ 0.92, 78.1%/85.9%/0.85, and 88.6%/91.2%/0.91, respectively (Table 5; Figures 3J–L).

To improve sensitivity, we combined rHA, vHA, and cHA (in other words, the diagnosis was based on any HA that was ≥ the cut-off value). As shown in Table 5, the sensitivity of a combined test was 92.6% when predicting anterior predominant prolapse by rHA ≥ 13.38, vHA ≥ 18.01, or cHA ≥ 8.42 cm^2^ (Figure 3M). The sensitivity was 92.8% when predicting apical predominant prolapse by rHA ≥ 14.65, vHA ≥ 18.12, or cHA ≥ 8.62 cm^2^ (Figure 3N). The sensitivity was 88.9% when predicting posterior predominant prolapse by rHA ≥ 14.79, vHA ≥ 20.38, or cHA ≥ 8.42 cm^2^ (Figure 3O).

Next, we combined BNM (≥20.50 mm for anterior predominant prolapse), CM (≥15.77 mm for apical predominant prolapse), or RAM (≥18.45 mm for posterior predominant prolapse), and the three HAs (where the diagnosis was based on either any HA or a compartment-specific mobility that was ≥ the cut-off value) (Table 5; Figure 3). This led to an improvement in the sensitivity from 92.6% (Figure 3M) to 94.9% (Figure 3P) when predicting anterior predominant prolapse by rHA ≥ 13.38, vHA ≥ 18.01, cHA ≥ 8.42 cm^2^, or BNM ≥ 20.50 mm. The sensitivity improved from 92.8% (Figure 3N) to 97.1% (Figure 3Q) when predicting apical predominant prolapse by rHA ≥ 14.65, vHA ≥ 18.12, cHA ≥ 8.62 cm^2^, or CM ≥ 15.77 mm. Finally, the sensitivity improved from 88.9% (Figure 3O) to 95.6% (Figure 3R) when predicting posterior predominant prolapse by rHA ≥ 14.97, vHA ≥ 2 0.38, cHA ≥ 8.42 cm^2^, or RAM ≥ 18.45 mm.

In parallel with an increasing sensitivity associated with combined ultrasound measurement, the negative predictive value (NPV) also increased significantly (Table 5).

## Discussion

Transperineal ultrasound is now used extensively for surveillance of pelvic floor function (18). For a disease as common and asymptomatic as POP (19, 20), the use of transperineal ultrasound as a routine front-line test may potentially enhance discovery and eliminate the inconveniences associated with the POP-Q. Transperineal ultrasound is also helpful to encourage vital pelvic floor training in a timely manner (21, 22). In the present study, we found that combining levator hiatal area and pelvic organ mobility significantly increased the sensitivity of transperineal ultrasound when diagnosing stage 2–3 prolapse. Our results may be explained by the pathophysiology of pelvic floor dysfunction. Female pelvic organ prolapse primarily develops after traumatic injury to the levator ani muscle (LAM) (8, 23). The LAM consists of the iliococcygeus, ischiococcygeus, pubourethralis, pubovaginalis, and puborectalis (the latter three muscles are also referred to collectively as the pubovisceral muscle). The first four muscles hold pelvic floor and pelvic organs at a normal position (the supportive portion of the LAM), whereas the puborectalis muscle exhibits a sphincteric function to keep the levator hiatus tight (the sphincteric portion of the LAM) (7, 8). The most frequently detected obstetric trauma is an avulsion injury to the supportive portion of the LAM (7, 8, 23); this is associated with increased pelvic organ mobility and downward displacement of the pelvic structures (12, 13). Trauma to the puborectal muscle is associated with an enlarged levator hiatus (8, 24, 25). Therefore, we hypothesized that by combining pelvic organ mobility and hiatal area, we may be able to improve sensitivity. In addition, the working schedule was highly convenient: first pelvic organ mobility was checked by 2-D ultrasound, then we transferred to 3-D ultrasound to measure the hiatus areas.

Numerous researchers have studied the validity of an enlarged levator hiatus measured by transperineal ultrasound for the prediction of POP. For example, Dietz (11) found that for Caucasian women, the appropriate cut-off was 25 cm^2^ for the Valsalva maneuver. In another study, involving women in Shanghai, Dou et al. (26) reported that the value of 19.5 cm^2^ on Valsalva was the most effective predictor. In the present study, carried out in Hebei province, we found that the cut-off values of hiatus area under the Valsalva maneuver were 18.01, 18.02, and 20.38 cm^2^, when predicting anterior-, apical-, and posterior-predominant POP respectively. Our results were similar to those reported by Xiao et al. (27) (19 cm^2^) for the prediction of stress urinary incontinence (SUI) among women in Guangzhou city in south China. Although Xiao et al. focused on SUI as their target disease, 85.8% of the POP cases in our present study also experienced SUI.

An abnormally larger bladder neck mobility is closely associated with SUI; cut-offs of 31.5, 15.5, and 24 mm have been reported previously for predicting this disease among Chinese women (28–30). Orn et al. (31) reported that this large variation may be caused by the confounding effect of levator co-activation. Xiao et al. (30) reported that the mean bladder neck mobility of 283 cases of SUI and 72 normal women were 29.7 ± 10.3 and 15.8 ± 6.6 mm, respectively. In our present study, these same parameters were 29.7 ± 12.6 and 13.8 ± 9.0 mm, respectively. Although these results are comparable, we reached a lower cut-off (19.65 mm) than Xiao et al.' s (30) (24.00 mm). Considering cases with unsuccessful Valsalva maneuver were excluded in both studies, other reasons for the discrepancy may be that we included age (over/under 50 years old) and body mass index (BMI) when constructing ROC analysis.

The strength of our study lies in the fact that all of the women included in this study were clinically examined according to the POP-Q system by one gynecologist who was blinded to ultrasound examination data; this reduces inter-observer misclassification. During enrollment, we included not only women with symptoms that were strongly suggestive of POP such as vaginal bulge or lumping, but also women with symptoms of lower urinary tract incontinence, pelvic pain, and chronic constipation. This may have helped to make our subjects more representative. To reduce the confounding effect imposed by other factors, we excluded cases with a history of hysterectomy, corrective pelvic floor surgery, stage 4 POP, and those who failed to perform a Valsalva maneuver.

Our study has some limitations that need to be considered. First, this was a single center study of Chinese women; our results now need to be verified on a larger scale in other centers and other ethnic groups. Second, we did not exclude subjects according to restrictions involving age or BMI. This may have affected our comparisons between cases and controls. Third, due to sample size, we did not analyze ultrasound parameters separately for the diagnosis of pelvic floor dysfunction according to specific symptoms, such as stress urinary incontinence, vaginal bulge, or fecal incontinence. Multi-centered studies with larger sample size is warranted.

## Conclusion

Combining measurements of levator hiatus and pelvic organ mobility improved the sensitivity of transperineal ultrasound for the diagnosis of POP. This may be used as a frontline test to facilitate POP-Q grading or to monitor the effect of pelvic floor exercise programs.

## Data Availability Statement

The raw data supporting the conclusions of this article will be made available by the authors, without undue reservation.

## Ethics Statement

The studies involving human participants were reviewed and approved by the study was approved by the Institutional Ethics Review Board of the Fourth Hospital of Hebei Medical University (20160203). A consent form was signed by all participants. The patients/participants provided their written informed consent to participate in this study.

## Author Contributions

XW was involved in study design, data collection, transperineal ultrasound, and helped to write the manuscript. HT was involved in data collection, POP-Q quantification, and manuscript writing. XY was involved in data collection and transperineal ultrasound. QS was involved in data collection, transperineal ultrasound, and manuscript writing. YD was involved in data collection and transperineal ultrasound. DW was involved in fund application, study design, data analysis, manuscript drafting and approval to the submission. All authors contributed to the article and approved the submitted version.

## Funding

This research was supported by the applied medical technology program in Hebei Province (Reference: G2015039).

## Conflict of Interest

The authors declare that the research was conducted in the absence of any commercial or financial relationships that could be construed as a potential conflict of interest.

## Publisher's Note

All claims expressed in this article are solely those of the authors and do not necessarily represent those of their affiliated organizations, or those of the publisher, the editors and the reviewers. Any product that may be evaluated in this article, or claim that may be made by its manufacturer, is not guaranteed or endorsed by the publisher.
